# Sperm epigenetics and sperm RNAs as drivers of male infertility: truth or myth?

**DOI:** 10.1007/s11010-024-04962-w

**Published:** 2024-05-08

**Authors:** Loredana Leggio, Greta Paternò, Fabrizio Cavallaro, Marco Falcone, Silvia Vivarelli, Claudio Manna, Aldo E. Calogero, Rossella Cannarella, Nunzio Iraci

**Affiliations:** 1 Department of Biomedical and Biotechnological Sciences (BIOMETEC), University of Catania, Catania, Italy; 2 Department of Biomedical and Dental Sciences, Morphological and Functional Imaging, Section of Occupational Medicine, University of Messina, 98125 Messina, Italy; 3 Department of Biomedicine and Prevention, University of Rome “Tor Vergata”, Rome, Italy; 4 Biofertility IVF and Infertility Center, Rome, Italy; 5 Department of Clinical and Experimental Medicine, University of Catania, Catania, Italy

**Keywords:** Spermatozoa, Male infertility, Epigenetics, Methylation, Sperm RNAs, MicroRNAs, Extracellular vesicles

## Abstract

Male infertility represents a complex clinical condition that often challenges the ability of reproductive specialists to find its etiology and then propose an adequate treatment. The unexplained decline in sperm count, as well as the association between male infertility and mortality, morbidity, and cancer, has prompted researchers toward an urgent need to better understand the causes of male infertility. Therefore, molecular biologists are increasingly trying to study whether sperm epigenetic alterations may be involved in male infertility and embryo developmental abnormalities. In this context, research is also trying to uncover the hidden role of sperm RNAs, both coding and non-coding. This narrative review aims to thoroughly and comprehensively present the relationship between sperm epigenetics, sperm RNAs, and human fertility. We first focused on the technological aspects of studying sperm epigenetics and RNAs, relating to the complex role(s) played in sperm maturation, fertilization, and embryo development. Then, we examined the intricate connections between epigenetics and RNAs with fertility measures, namely sperm concentration, embryo growth and development, and live birth rate, in both animal and human studies. A better understanding of the molecular mechanisms involved in sperm epigenetic regulation, as well as the impact of RNA players, will help to tackle infertility.

## Introduction

Couple infertility represents a significant public problem that burdens on the health, psychological, economic, and social aspects of couples of childbearing ages. According to the World Health Organization (WHO), as many as 48 million couples were diagnosed as infertile in 2010 [1], and nowadays, the prevalence may be even higher. A male factor occurs in about half of couples with infertility. It is usually associated with abnormalities in conventional sperm parameters (i.e., low sperm concentration, total sperm count, progressive or total motility, normal morphology, and viability). The prevalence of male infertility is steadily increasing worldwide. A meta-regression analysis of thousands of patients from around the world has shown a ⁓50% decrease in sperm concentration and total sperm count over the past forty years, apparently without any explanation [2]. It is worrying that the global decline continues through the twenty-first century at an accelerated pace [2]. The decline in the sperm number is also associated with a higher prevalence of idiopathic forms than in the past. In fact, in 72% of a cohort of more than 26,000 patients who referred to an Andrology center for infertility, no cause was found to explain the abnormality sperm parameters, although the patients underwent a complete diagnostic work-up [3]. Similarly, a prospective study of 1,737 infertile subjects reported a prevalence of idiopathic oligozoospermia in 75% of cases [4]. If we also consider, as it has happened especially in recent years, that poor sperm quality is associated with a greater risk of hospitalization, cardiovascular disease, diabetes, mortality, morbidity [5, 6], and cancer [7], the compelling need to better understand the etiology of male infertility and its proper treatment is easily comprehensible.

The aforementioned reasons have led several researchers to study biofunctional sperm parameters [8]. However, their evaluation is often insufficient to identify the underlying causes of male infertility [9]. To overcome this pitfall, molecular biologists have implemented studies on the epigenetic origin of both male infertility and embryo development abnormalities [10, 11]. Epigenetics focuses on all the changes occurring during meiosis/mitosis that regulate gene expression without modifying the DNA sequence. These studies have allowed in some cases to better understand the molecular mechanisms behind the so-called idiopathic infertility. DNA methylation and post-translational modifications (PTMs) of histones represent the major epigenetic changes occurring at the sperm level [12]. Also, sperm cells, traditionally thought to be transcriptionally inert, have been found to contain various RNA species, with a previously hidden role in fertilization and early embryonic development [12]. For this reason, this narrative review article aims to thoroughly and comprehensively evaluate the relationship between sperm epigenetics and human fertility.

## Overview of sperm epigenetics and sperm RNAs

Formation of a mature spermatozoon requires mitotic proliferation of spermatogonia with meiotic divisions and morphological differentiation of spermatids. This leads to the generation of highly specialized cells characterized by the presence of a head, an intermediate portion and a flagellum or tail. The specific organization of spermatozoa is required to cross the hostile female reproductive tract, penetrate the oocyte, and ultimately complete multiple post-penetration events [13]. They allow sexual reproduction through their union with the female oocyte during fertilization. For this purpose, spermatozoa must maintain their structure and DNA integrity during their journey toward the oocyte. They are “stripped-down” cells, without typical organelles but with a long and strong flagellum, to propel them through an aqueous medium [14, 15]. The sperm head contains secretory vesicles called “acrosomal vesicles” enriched with hydrolytic enzymes that may help spermatozoa to penetrate the outer coat of the oocyte. The tail is the structure that allows sperm motility and, therefore, is rich in mitochondria [16, 17].

Additionally, the sperm head contains a condensed haploid nucleus with highly packed DNA to facilitate motility during fertilization. In this context, epigenetic processes, which include DNA modifications (5-methylcytosine, 5mC, and 5-hydroxymethylcytosine, 5hmC) and histone PTMs (acetylation, methylation, phosphorylation, ubiquitination, etc.), play a crucial role in the complex regulation of gene expression [18]. The sperm epigenome is extremely variable, with fluctuations over time based on specific environmental cues [13]. The modifications acquired during spermatogenesis allow significant reorganizations of the structure and maturation of sperm chromatin. Therefore, spermatogenesis is particularly vulnerable to epigenetic alterations that can result in spermatogenic abnormality and infertility [19]. During spermatogenesis, approximately 90–95% of all chromatin histones are replaced by small nuclear proteins enriched in arginine domains, called protamines [13], in a process known as “protamination”. The histone-to-protamine transition tightly compacts DNA and causes gene repression, while also decreasing susceptibility to external stimuli. The remaining 5–10% of histones can also be modified [20], further showing the crucial contribution of epigenetic events which, if altered during spermatogenesis [21], could impact sperm count and embryo quality [22].

Recently, sperm RNAs have been shown to play an important role in this context [12]. Indeed, spermatozoa contain coding and non-coding RNAs (ncRNAs), both long and small, such as microRNAs (miRNAs), Piwi-interacting RNAs (piRNAs), small interfering RNAs (siRNAs), tRNA-derived fragments (tRFs), circular RNAs (circRNAs), and others [23–27]. These RNAs are involved in sperm maturation, transmission of paternal phenotypes, and embryo development. Any changes in the amount or composition of sperm RNAs may cause abnormalities in spermatogenesis, although the exact molecular mechanisms need to be further elucidated [28]. Overall, these aspects represent a breakthrough in the field of human reproduction and infertility [29].

**Fig. 1:**
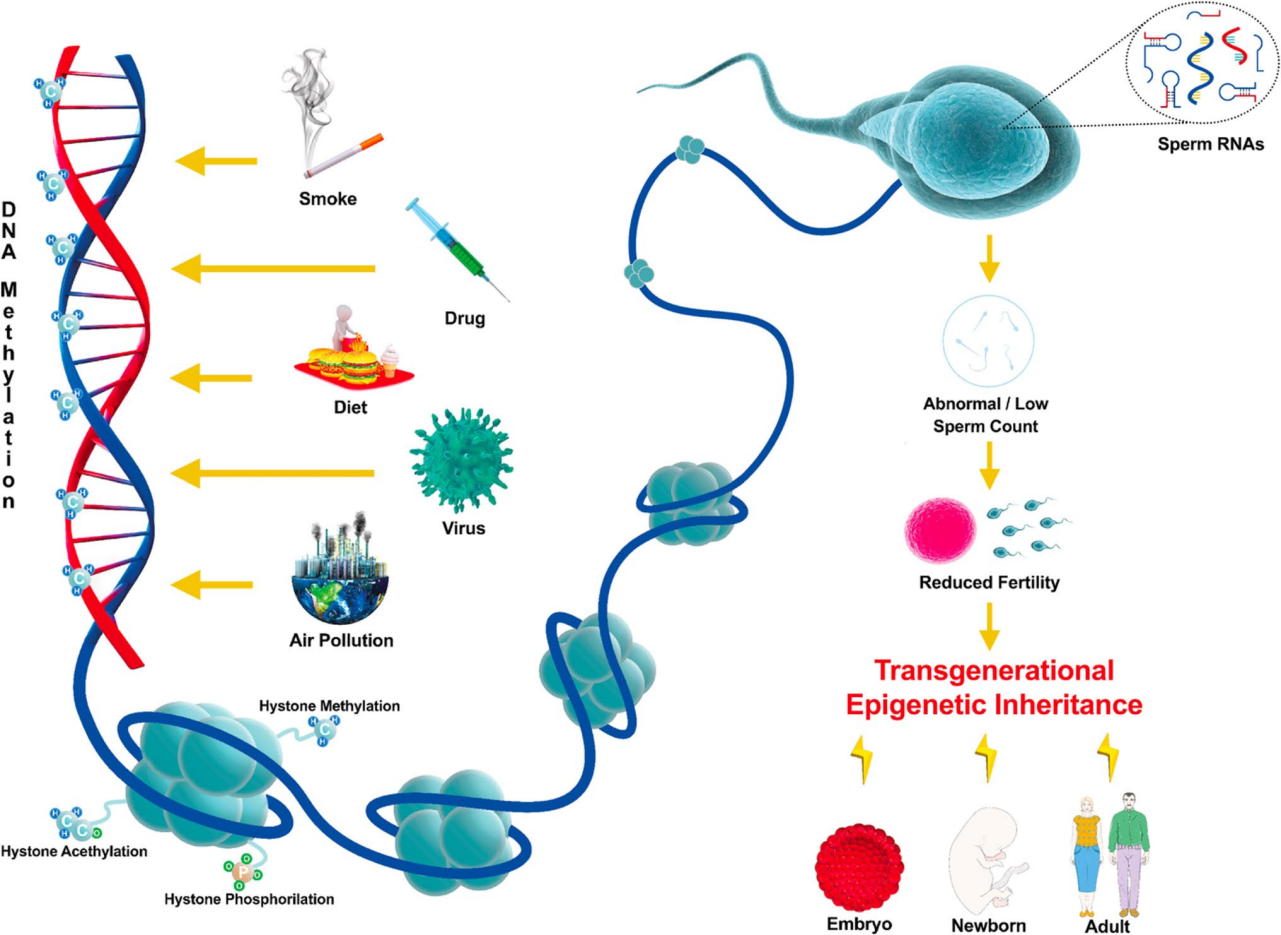
External factors (e.g., smoking, drugs, diet, viruses, air pollution) may influence the epigenetic modifications and RNAs of spermatozoa with possible consequences on fertility and offspring outcomes

Here, we will first focus on the methods used to study epigenetics and transcriptomics in spermatozoa. Indeed, the choice of the most appropriate technology is of particular importance in the search for the strongest candidate markers in the context of the gene expression program in spermatozoa. Next, we will review evidence from animal and human studies showing how specific epigenetic modifications and alterations in RNA levels in spermatozoa are crucial during their maturation. Finally, the correlation with important parameters of sperm quality (count, volume, and morphology), embryo development, and disease onset in the offspring will be further discussed (Fig. 1) [29].

## Experimental approaches for sperm retrieval

Seminal fluid is a heterogeneous sample, in which different cell types can be found. These include leukocytes, epithelial cells, immature germ cells, and spermatozoa. The procedures used to separate spermatozoa from other cell types represent a key methodological aspect in examining DNA methylation and RNAs specifically in the sperm population. Indeed, incomplete separation procedures lead to the analysis of a sample contaminated with other cell types and, consequently, to biased results.

In this review, studies conducted on animals and humans were carefully selected. Most animal research has obtained spermatozoa by epididymal dissection [30–39] and separating them using a swim-up protocol. This method allows for the separation of motile spermatozoa from other cell types in the ejaculate. In particular, this procedure has been employed in studies on small animals. In more detail, spermatozoa were obtained from the caput in [34] and from the cauda epididymis in [30, 35–39] of the epididymis, the latter allowing the collection of more mature spermatozoa. The remaining articles did not specify the exact part of the epididymis from which spermatozoa were obtained. Studies conducted on large animals (e.g., bulls or boars) obtained spermatozoa directly from ejaculate [40, 41]. However, the procedures used to separate spermatozoa from other cell types are not mentioned, which is a reason to take the results with caution. In one case, immature spermatozoa were isolated directly from the testis [42] after digestion with collagenase and anti-trypsin. Finally, few studies included in this review did not mention how spermatozoa were separated [43, 44].

On the other hand, most human studies have retrieved spermatozoa from the ejaculate and used sperm separation protocols, such as swim-up [45–47], centrifugation gradient [48–56], or a combination of the two methods [57, 58]. These approaches allow the collection of only motile spermatozoa. A minority of studies have incubated samples with somatic cell lysis buffer (SCLB) which, by lysing specifically the membranes of this type of cells, allows the collection of both motile and immotile spermatozoa from the ejaculate. Only 2 studies did not use any procedure to separate sperm cells, and these results should be taken with caution [59, 60].

## Experimental approaches for sperm epigenetics studies

Nucleic acids can be extracted from several sources, including isolated spermatozoa and seminal fluids, through a variety of methods, each specifically chosen to perform the intended downstream application. In this context, the most employed method for DNA purification has been phenol–chloroform-based extraction [31, 38, 42, 45–49, 51, 58, 60, 66, 67] through a separation into two phases. Other studies [31, 33, 34, 43, 52, 55, 59, 68, 69] have used solid phase extraction on silica matrices, which allows for higher purity in less time. Furthermore, a complementary DNA hybridization with functionalized magnetic beads was used, avoiding any centrifugation [35].

As the field of epigenetics is rapidly expanding, interest in exploring new technologies to decode epigenetic landmarks in both health and disease states has increased dramatically. To obtain reliable results, it is crucial to choose the protocols to evaluate the methylation status, the PTM of histones, as well as the characterization of sperm RNAome. The pros and cons of each technique are highlighted in Table 1. The specific results obtained in each study will be further discussed in the next Sections and in Tables 2 and 3.

**Table 1:** Description of the techniques used in the reviewed studies

Target	Principle	Specificity	Strategy	Pros	Cons	References
DNA methylation	Bisulfite treatment - selectively deaminates unmodified C to U while leaving 5mC and 5hmC intact	Locus	MS-PCR and Sanger-Seq	Ease of design and execution, sensitivity in the detection of the methylation status in the specific loci of interest, screen a large number of samples	Low throughput, variability and false-positive result, few CpG sites can be detected	[31–35, 38, 42, 45, 46, 51, 57–59, 66, 67, 72]
			HRM	High sensitivity, small DNA input required, high capability of analyzing multiple CpG sites in a region	Low specificity, sensitivity is strongly affected by the primer design and annealing temperatures	[59]
			COBRA	More than one restriction site can be tested on one PCR product, given that additional sites are available	Time-consuming protocol, limitation by available restriction sites, false-positives by incomplete digestion	[35]
		Array	Illumina HumanMethylation 27 DNA Analysis Bead Chip	High sensitivity and reproducibility, high-throughput, requires small amount of input DNA (only 1 μg)	Cross-reactive probes, SNP-affected probes, Array bias (batch effects)	[48]
			Illumina Infinium HumanMethylation450 BeadChip	High coverage (≥ 96% of CGIs), high sensitivity, high reproducibility, high-throughput	Complex data analysis and normalization, combines two assays that interrogate different number of CpGs, cross-reactive probes	[68]
			Infinium Human MethylationEPIC BeadChip microarrays	Ease of use, time- and cost-effective, good agreement with DNA methylation measurements from other platforms	Human samples only, substantial degradation after bisulfite treatment, coverage is highly dependent on array design	[69]
		Genome	RRBS	High CGI coverage, suited for reduced-complexity obtained by bisulfite treatment, enriches for CpG-containing regions, cost-effective compared to WGBS, less data congestion	Less reproducible, lower coverage at intergenic and distal regulatory elements, substantial DNA degradation after bisulfite treatment, limited to regions in proximity to restriction enzymes’ recognition sites	[32]
			WGBS	Evaluate methylation state of almost every CpG sites, including distal regulatory elements and intergenic regions	High-cost, substantial DNA degradation after bisulfite treatment, cannot discriminate between 5mC and 5hmC	[43]
	Affinity enrichment of methylated regions using antibodies for 5mC	Genome	MeDIP	Cost‐effective, no mutations introduced, specific to 5mC/5hmC depending on the antibody, more sensitive in regions with low CpG density	Biased toward hypermethylated regions, does not identify individual 5mC sites, inability to predict absolute methylation level	[33, 67]
	Others	Global methylation patterns	HPLC	Fast and sensitive	Not specific, unable to discriminate regions of interest	[49, 72]
			FC	Can analyze large number of cells		[49]
			IF/ICC	High sensitivity		[49, 58]
			ELISA	Can discriminate between 5mC and 5hmC		[52]
			MALDI-TOF–MS	Can discriminate between 5mC and 5hmC		[34]
Chromatin Modifications	Chromatin Immunoprecipitation - antibodies that recognize nucleosome complexes with a given PTM (e.g., H3K9me1/2/3, H3K27ac)	Genome	NChIP	Use of native chromatin, ChIP is very efficient, specificity of the antibody binding to unfixed chromatin is more predictable	Not applicable to non-histone proteins, danger of protein rearrangement during chromatin preparation and precipitation, selective digestion of particular chromatin domains during preparation may occur	[43]
RNA	Reverse-Transcription	Locus specific qPCR	SYBR	Powerful, fast, cost-effective	Results are dependent on primer efficiency and product length	[41, 53, 55, 71, 72]
			TaqMan Probes	Accurate quantification, highly specific	High-cost	[30, 54]
		Array	TaqMan Array MicroRNA qPCR	High-throughput, wide expression screening	High-cost	[56, 71]
		Transcriptome (several methods for library construction)	RNA-Seq	Identifies all RNA species, high sensitivity, high-throughput, enables population studies	Complex data analysis, requires specific custom algorithms to discriminate putative sncRNAs, possible bias for some RNAs	[30, 36, 38–41, 47, 60, 70–72]

**Table 2:** Sperm epigenetics and sperm RNAs: evidences in animals

		Authors and year	Exposure/condition	Target	Impact on fertility	Starting tissue	Principle of analysis	References
Animal models	Sperm epigenetics	Doshi et al., 2011	BisPhenol A (BPA)	Alteration of estrogen receptor promoter and enhancement of DNMT expression	Impairments in spermatogenesis	Testis	MS-PCR and Sanger-seq	[66]
		Xu et al., 2013	Tobacco smoke	Proteins of testes involved in both signaling and metabolic pathways	Impairments in spermatogenesis	Spermatocytes and round spermatids obtained after testis digestion	MS-PCR, Sanger-seq, MALDI-TOF	[42]
		Lambrot et al., 2013	Folate deficiency	Genes implicated in development, diabetes, autism and schizophrenia	Musculoskeletal and craniofacial malformations in offspring	Spermatozoa from caput epididymis	MeDIP-chip array, MS-PCR and Sanger-seq, MALDI-TOF–MS	[34]
		Ge et al., 2014	Diabetes and/or obesity	DNA methylation in Peg3 and H19	Implication for testis weight, number of Leyding and Sertoli cells, number of spermatogonia	Spermatozoa from caput epididymis	COBRA and Sanger-seq	[35]
		Öst et al., 2014	Diet	Increased expression of heterochromatic-embedded metabolic genes (characterized by active deposition of H3K9/K27me3	Implications for chromatin state in both mature sperm and offspring embryos	Spermatozoa from seminal vesicles	TruSeq stranded sample preparation kit for Illumina RNA-seq	[70]
		Prados et al., 2015	Di-(2-ethylhexyl)phtalate (DEHP)	Increase in hyper-methylation of genes involved in both sperm chemotaxis and post-transcriptional regulatory mechanism in the C57BL/6J versus FVB/N	Impairments in spermatogenesis	Spermatozoa from vas deferens and epididymis	MBD-Seq on Illumina HiSeq2000, MS-PCR and Pyrosequencing	[33]
		Dai et al., 2016	Tobacco smoke	Hyper-methylation at the level of Sord gene promoter	Impairments in sperm maturation and capacitation	Spermatozoa from epididymis	MS-PCR, Sanger-seq, MALDI-TOF	[31]
		Murphy et al., 2018	Cannabis/tetrahydrocannabinol (THC) smoke	⁓6,640 CpGs sites abnormally methylated and involved in the Hippo signaling and Cancer pathways	Abnormal embryonic development	Spermatozoa from epididymis	RRBS on Illumina HiSeq1500/2500, MS-PCR and Pyrosequencing	[32]
		Sadler-Riggleman et al., 2019	Vinclozolin and dichlorodiphenyltrichloroethane (DTT)	Transgenerational alterations in DNA methylation, non-coding RNAs, and gene expression in rat Sertoli cells	Testicular disease; decreased sperm count and/or motility in offspring	Sertoli cells obtained after testis digestion	MeDIP-Seq on Illumina HiSeq 2500	[67]
		Liu et al., 2019	Carbendazim and chlorothalonil	Modulation of estrogen receptor signaling pathway and abnormal DNA and histone methylation	Decrease in spermatozoa concentration and motility	Spermatozoa (isolation not mentioned)	NChIP-Seq on Illumina HiSeq 2500 and WGBS on Illumina HiSeq X10	[43]
	Sperm RNAs	Rodgers et al., 2015	Stress	miRNAs	Offspring with altered stress response	Zygotes	EvaGreen Supermixon with DELTA gene assays qPCR, Transcriptomics on TruSeq Stranded mRNA Kit with poly-A enrichment on Illumina HiSeq2000, TaqMan RT-qPCR Array for microRNA	[71]
		Chen et al., 2016	High-fat diet	tRNA involved in metabolic pathways and metabolic disorders	Metabolic disorders in offspring	Spermatozoa (isolation not mentioned)	Transcriptomics of Mature-sperm-enriched small RNA, Sequencing platform not specified	[44]
		Sharma et al., 2016	Low protein diet	Higher level of tRNA-Gly-CCC, -TCC, and -GCC; Up-regulation of tRF-Lys-CTT and tRF-His-GTG	Impairments in embryo development	Spermatozoa from cauda epididymis and rete testis	Sequential rounds of PCR and Illumina HiSeq 2000 for small RNAs, TaqMan microRNA assays for tRF, RNA-seq with SMART-Seq on Illumina HiSeq 2000	[30]
		Capra et al., 2017	–	miRNA and piRNA altered expression between low-motility versus high-motility bovine spermatozoa, with more piRNAs in highly motile cells	Impairments in spermatogenesis and sperm motility	Spermatozoa from bull's ejaculates	Transcriptomics on Illumina Truseq Small RNA Preparation kit and Illumina HiSeq 2000	[40]
		Guo et al., 2017	–	RNA-depleted spermatozoa	Impairments in embryo development	Spermatozoa from cauda epididymis	Sequencing on Illumina Hiseq 2500	[36]
		Zhang et al., 2018	High-fat diet	Deletion of Dnmt2 reduced the levels of m5C and m2G of small RNAs and altered their expression profile	Prevention of metabolic disorders in offspring	Spermatozoa from cauda epididymis and vas deferens	SYBR green PCR on LightCycler 480 and TruSeq Stranded mRNA Library kit on Illumina HiSeq 4000 for total RNA, SYBR qPCR for sncRNAs, MS-PCR and Pyrosequencing for DNA, Agilent 6460 Triple Quadrupole-Agilent 1200 HPLC for MS-HPLC	[72]
		Conine et al., 2018	Small RNAs from the caput portion	Gene expression analysis of mature vs. immature spermatozoa	Impairments in embryo development	Spermatozoa from cauda epididymis	SMART-Seq on NextSeq 500	[38]
		Godìa et al., 2020	–	circRNAs involved in spermatogenesis, cilium assembly, and developmental processes in pigs	Altered sperm quality	Spermatozoa from boars’ ejaculates	SMARTer Library prep and Sequencing on Illumina HiSeq 2000/2500 for total RNA, NEBNext Library Prep Kit and Illumina HiSeq 2000 for sncRNAs, qPCR and SYBR Select Master Mix and Sanger Sequencing for circRNAs	[41]
		Tyebji et al., 2020	Toxoplasma infection	Alteration of the sperm small ncRNA profiles	Altered sperm quality; impairments in embryo development	Spermatozoa from cauda epididymis	NEBNext Multiplex Small RNA Library Prep, qPCR for quantification and sequencing on Illumina HiSeq 2500	[39]

**Table 3:** Sperm epigenetics and sperm RNAs: evidences in humans

		Authors and year	Exposure/condition	Target	Impact on fertility	Method for sperm collection	Principle of analysis	References
Human modifications	Sperm epigenetics	Marques et al., 2004	–	MEST and H19	Impairments in spermatogenesis; altered sperm quality	Gradient centrifugation followed by swim up	MS-PCR and Sanger-seq	[57]
		Poplinski et al., 2010	–	IGF2/H19 control region 1 and MEST	Alterations in sperm count and motility	Swim up	MS-PCR and Sanger-seq	[45]
		Wu et al., 2010	–	MTHFR	Azoospermia and infertility	Swim up	MS-PCR and Sanger-seq	[46]
		Pacheco et al., 2011	–	HDAC1 and DNMT3A	Altered sperm motility	Percoll gradient	Illumina Infinium HumanMethylation27 BeadChip Array	[48]
		Marques et al., 2011	–	H19 and MEST; DNMT1, DNMT3A-B	Impairments in spermatogenesis	Gradient centrifugation followed by swim up	MS-PCR and Sanger-seq, IF	[58]
		Barzideh et al., 2013	–	Analysis of global methylation status	Impairments in spermatogenesis; altered sperm quality	Percoll gradient followed by anti-CD45-guided leukocyte removal	HPLC, FC, IC	
		Tian et al., 2014	–	LINE1, BRDY and MTHFR	Altered sperm quality and motility	Wash plus centrifugation	HRM, MS-PCR and Sanger-seq	[59]
		Laqqan et al., 2017	/	PRICKLE2, ALS2CR12, ALDH3B2, and PTGIR	Altered sperm quality and motility	Gradient centrifugation followed by SCLB incubation	Bisulfite and Infinium 450 K BeadChip array	[50]
		Dong et al., 2017	Tobacco smoke	H19 and SNRPN	Altered sperm count and motility	Percoll gradient	MS-PCR and Pyrosequencing	[51]
		Santana et al., 2020	Varicocele	Analysis of global methylation status	Altered sperm quality and motility	SCLB incubation	Bisulfite and Infinium Human MethylationEPIC BeadChip microarrays	[69]
		Cheng et al., 2022	Air pollution	Analysis of global methylation status	Altered sperm count and motility	Percoll gradient	MethylFlashTM Global DNA Methylation (5 mC) ELISA Kit	[52]
	Sperm RNAs	Cui et al., 2015	–	miR-34c	Congenital malformations and alteration of sperm quality	Density gradient	miScript SYBR Green RT-qPCR	[53]
		Savadi-Shiraz et al., 2015	–	PRM1, PRM2, and TNP2	Altered sperm quality	Density gradient	AmpliTaq Gold RT-qPCR	[54]
		Donkin et al., 2016	Obesity	piRNA targets involved in food intake regulation	Epigenetic inheritance of metabolic disorders	Swim up	sncRNA-seq	[47]
		Sadakierska-Chudy et al., 2020	–	AKAP4, PTK7 PLCζ, and POU5F1	Abnormal embryonic development	Gradient centrifugation	TaqMan low-density array gene expression	[56]
		Amor et al., 2021	Tobacco smoke	H2BFWT, PRM1, PRM2, TNP1, and TNP2	Protamine deficiency and DNA fragmentation	Gradient centrifugation followed by SCLB incubation	SYBR Green qPCR	[55]
		Hamilton et al., 2022	–	miR-4755-3p, miR-92a-3p, 5’-tRF-Asp-GTC; 5’-tRF-Phe-GAA, let-7f-2-5p	Difference in blastocyst formation rate	Wash plus centrifugation	Transcriptomics on NextSeq 550 Sequencing System	[60]

5mC is the predominant DNA modification and accounts for approximately 5% of all cytosines. At a gene-specific resolution, several studies have used methylation-specific PCR (MS-PCR) on bisulfite-converted DNA [31–35, 38, 42, 45, 46, 51, 57–59, 66, 67, 72]. In this way, after unmethylated cytosines are deaminated to uracils, it is possible to determine the methylation status at the specific loci of interest. Another locus-specific method was applied by [59], through high-resolution melting (HRM) analysis. This technique can detect single base pair differences by their distinct melting curves, after DNA treatment with sodium bisulfite. Another approach integrates bisulfite conversion-based PCR with restriction digestion, namely combined bisulfite restriction analysis (COBRA), as used in [35].

Followed by bisulfite conversion, the global DNA methylation status can also be determined by hybridization-based microarrays, as in [48, 50, 69]. The HumanMethylation27 DNA Analysis Bead Chip, Illumina HumanMethylation450 (HM450K), and Infinium Human MethylationEPIC BeadChip microarrays could interrogate 27.578, 450.000, and 935.000 CpG sites, respectively [48, 50, 69]. These array technologies have been widely used due to their high versatility, focusing on the most biologically significant regions of the genome. Further scale-up to methylome-wide studies were applied in [32, 43]. In particular, the authors in [32] used reduced representation bisulfite sequencing (RRBS) size fractionation of DNA fragments, after digestion with BglII or MspI. These enzymes enrich for CpG-containing segments, without targeting specific regions in the genome. Finally, whole-genome bisulfite sequencing (WGBS) was applied to cover all CpG sites, for a comprehensive methylome profile [43], also used by major epigenome consortia (e.g., ENCODE).

However, bisulfite treatment has significant drawbacks, being a very harsh chemical reaction that may cause DNA damage and loss [73]. Furthermore, converted unmodified cytosine (95% of all cytosines in the genome) leads to reduced DNA sequence complexity, lower mapping efficiency, and biased genomic coverage [74]. Therefore, two studies [33, 67] used different approaches, such as methyl binding domain-seq (MBD-Seq) and methylated DNA immune-precipitation (MeDIP-Seq) [33, 67]. They specifically enriched methylated regions using antibodies for 5mC, followed by high-throughput DNA methylation profiling [33, 67].

Some studies have used alternative methods for methylation analysis [34, 49, 52], such as high-pressure liquid chromatography (HPLC), flow cytometry (FC), immunofluorescence (IF), enzyme-linked immunosorbent assay (ELISA), and matrix-assisted laser desorption/ionization–time of flight mass spectrometry (MALDI–TOF–MS) [34, 49, 52]. However, these methods are nonspecific, lacking the possibility to differentially characterize regions of interest, and only cover global methylation patterns.

Another crucial epigenetic mechanism regulating gene expression occurs through the PTM of histone N-terminal tails. So far, only one study considered the role of histone modifications in the context of sperm epigenetics [43]. The authors used native chromatin immunoprecipitation followed by NGS (NChIP-Seq). This strategy is based on the selection by immune recognition of naturally occurring protein-DNA interactions, in particular, for histone modifications [43].

## Experimental approaches for sperm RNA analysis

Regarding RNA extraction, most studies used an acid-guanidinium-phenol-based strategy [28, 30, 32, 36, 38–42, 53, 56, 67, 70–72]. Similar to DNA extraction, paramagnetic beads can be applied for total RNA extraction, a method used in [38]. Subsequently, quantitative real-time PCR (qPCR) has been widely applied to study sperm RNAs, both in animal and human research [30, 41, 53–55, 71, 72]. Some studies have used SYBR dye [41, 53, 55, 71, 72]. While an economical and simple strategy, SYBR data can depend on primer efficiency and product length and these limits must be carefully considered. The use of TaqMan technology can improve the specificity of qPCR, as in [30, 54]. This is also useful for scale-up approaches, as in [56, 71], where a high-throughput TaqMan qPCR array was applied to profile a variety of selected targets.

The remaining studies [30, 36, 38–41, 47, 60, 70–72] applied a broader analysis to comprehensively profile all RNA species in spermatozoa, via RNA-seq. This technique can also detect new sequences, ranging from mRNAs to miRNAs, piRNAs, and circRNAs. In particular, one study [40] used a specific bioinformatics pipeline to identify piRNAs and miRNAs among bull sperm samples [40].

A peculiar strategy can be applied to circRNAs. Due to their unique structure, these can be retro-transcribed and amplified by qPCR to then undergo Sanger sequencing, similar to the sequencing of cloning vector [41].

One study [28] did not specify the technology for RNA analyses, so it should be taken with caution [28].

## Animal evidence: impact of sperm epigenetic mechanisms 

The establishment of specific genomic methylation patterns plays a pivotal role in the so-called hereditary silencing, regulating germ cell development [75]. Indeed, these patterns occur in two different moments, one in the primordial germ cells (PGCs), and one during preimplantation for transmission to offspring [76].

One of the first studies that described the important role of DNA methylation for offspring viability dates back to 1992, when Li and colleaguesinduced targeted mutations in the Dnmt gene in the germline of mice, resulting in abnormal embryo development and increased lethality of the embryo [77]. DNA methylation is required during meiosis for the production of male germ cells and, after fertilization, for embryo development. However, after fertilization, the paternal genome undergoes demethylation, except for imprinted genes and repeated sequences [78]. During spermatogenesis, specific environmental cues can influence the correct DNA methylation process inducing sperm alterations and infertility [79]. In particular, paternal lifestyle can alter epigenetic marks in the germline, resulting in the alteration of both spermatozoa and offspring [80]. Incomplete or abnormal chromatin condensation causes DNA damage and consequent changes in several sperm parameters, such as morphology (teratozoospermia), progressive motility (asthenozoospermia), and concentration (oligozoospermia), with possible effects on fertility and embryo development [81]. Although the exact mechanisms responsible for the aberrant sperm DNA methylation in male infertility need to be deeply characterized, both environmental factors (e.g., pesticides and other toxicants) and lifestyle habits (such as smoking, alcohol consumption, and diet) may have an overriding influence [82, 83].

Tobacco smoke is the most common factor known to have a strong negative impact on sperm DNA methylation, but the precise mechanisms by which paternal smoking is associated with detrimental effects on fertility and sperm parameters are poorly understood. In 2013, Xu and coworkers investigated the protein profile of testes of mice exposed daily to cigarette smoke, using the MALDI-TOF–MS analysis [42]. The authors found that during spermatogenesis, exposure to cigarette smoke caused a change in the testicular proteome, particularly in signaling and metabolic pathways with consequent impairments on spermatogenesis [42]. Dai and collaborators in 2016 obtained similar results [31]. They evaluated the protein profile in nicotine-exposed mouse epididymal tissue by two-dimensional gel electrophoresis and MS analyses. The results showed that there were proteins mainly involved in molecular transportation networks and the polyol pathway, indicating an impairment of the secretory functions of the epididymis. Furthermore, they found that nicotine exposureinduced hypermethylation of the promoter region of the *Sord* gene (sorbitol dehydrogenase), inducing reduced secretory function of the epididymis and thus preventing proper sperm maturation and capacitation [31]. Also, cannabis exposure has been reported to impact sperm methylome integrity in both human and rat models, as described by Murphy et al. in 2018 [32]. The authors analyzed the epigenetic profile using the RRBS approach, finding at least 6,640 CpGs sites whose methylation status was altered as a results of cannabis or THC exposure. These included genes in the Hippo signaling and cancer pathways, possibly implicated in growth regulation and consequent non-viable embryos [32].

Also, many environmental contaminants can alter the epigenetics landscape of male germ cells, thus posing a major threat to mammalian development. For instance, in 2011, Doshi and colleagues reported that exposure to bisphenol A (BPA), an estrogenic endocrine disruptor commonly used in the manufacture of polycarbonate plastics and epoxy resins, affects the epigenetic signature in testis, and consequently the health of offspring [66]. Notably, neonatal exposure to BPA alters the methylation of the estrogen receptor promoter and enhances the expression of DNMT3A and DNMT3B in adult rat testis, at both transcript and protein levels, supporting an aberrant DNA methylation at several gene loci that influence spermatogenesis and consequently fertility [66]. Similarly, Prados and colleagues in 2015, demonstrated that Di-(2-Ethylhexyl)-phthalate (DEHP), an industrial plasticizer commonly present in the environment, increases DNMT1 expression and, consequently, DNA methylation in the testis of mice, with effects on spermatogenesis, depending on the mouse strain [33]. Exposure to DEHP increased hyper- and decreased hypo-methylation in C57BL/6J vs. FVB/N mice. The same trend was observed at the level of gene promoters involved in both sperm chemotaxis and post-transcriptional regulatory mechanisms, with a more pronounced hypermethylation in the C57BL/6J strain [33]. In 2019, a study by Sadler-Riggleman et al. investigated the effects of exposure to environmental toxicants on the transgenerational inheritance of epigenetic marks [67]. In particular, their results demonstrated that transgenerational alterations in DNA methylation, ncRNAs and gene expression occurred in Sertoli cells exposed to the pesticides vinclozolin and dichlorodiphenyltrichloroethane (DDT). This suggests that germline exposure to environmental factors causes epigenetic and transcriptome alterations that can be transmitted to the next generations with varying outcomes, including testicular disease and a decreased sperm count and/or motility [67]. Similarly, several fungicides such as carbendazim and chlorothalonil, have been associated with aberrant DNA methylation [84]. Liu and coworkers demonstrated in their findings published in 2019, that low doses of each compound influence the spermatogenesis of pubertal mice, causing a decrease in sperm concentration and motility. In particular, these compounds act via modulation of estrogen receptor signaling, disrupting both DNA and histone methylation [43, 85].

In modern society, several diet-related diseases result from the influence on sperm epigenome [86]. In 2013 Lambrot and colleagues demonstrated that paternal diet is associated with birth defects in mice. In particular, sperm from folate-deficient mice showed differential DNA methylation of genes implicated in development, diabetes, autism and schizophrenia [34]. There is also evidence that pregestational diabetes and/or obesity impair DNA methylation in offspring spermatozoa, as demonstrated by Gen and colleagues in 2014. These spermatozoa have altered DNA methylation in *Peg3* and *H19* genes, with possible implications for testicular weight, Leydig and Sertoli cell number and spermatogonia number [35]. The same year, Ost and colleagues, observed that paternal diet influences chromatin status in both mature spermatozoa and offspring of Drosophila. High sugar intake increased the expression of heterochromatic-embedded metabolic genes (characterized by active deposition of H3K9/K27me3, as per ENCODE data), reprogramming offspring metabolism [70]. Also, a similar mechanism could regulate obesity susceptibility in mice and humans [70] (Table 2).

However, further studies are needed to better elucidate the mechanisms affecting sperm DNA methylation and their impact on male infertility. An improved knowledge of sperm epigenetic status in relationship with reduced reproductive capacity could become a new diagnostic and prognostic parameter to evaluate male infertility and pregnancy outcome, respectively.

## Animal evidence: impact of sperm RNAs 

New evidences support the presence of both coding and ncRNAs in spermatozoa, with functional roles in embryo growth and development, currently under intense investigation [87, 88].

The discovery of RNA in animal spermatozoa dates back to the 1980. Since then, it has been proposed that sperm RNAs play three possible functions, with poorly understood molecular mechanisms: (i) sperm maturation in the epididymis; (ii) transmission of the acquired phenotype from parents to offspring; and (iii) embryo development [89, 90]. In 2015, sperm miRNAs from a mouse model of chronic stress were found to be responsible for developing offspring with impaired stress responses, like their fathers [71].

Regarding the impact on sperm parameters, in 2017 Capra and co-workers characterized the small ncRNA content (both miRNAs and piRNAs) in cryopreserved bovine semen from a single animal, by RNA-seq [40]. They observed more piRNAs clusters in low- vs. high-motility spermatozoa. Similarly, miRNA targeting pathways related to cell apoptosis and alteration of spermatogenesis—which could affect sperm motility and therefore bull fertility—were found dysregulated in the low-motile fraction [40]. More recently, in 2020, Godìa and colleagues analyzed the circRNAs in 40 porcine ejaculates [41]. GO enrichment analysis of genes harboring circRNAs highlighted epigenetic functions, spermatogenesis, cilium assembly and developmental processes. Finally, the authors validated correlations between circRNAs and sperm motility, suggesting their important roles in sperm parameters, and consequently in infertility [41].

Turning to the consequences of sperm RNAs on embryo development, Chen and colleagues in 2016 demonstrated how a paternal high-fat diet (HFD)induced changes in the expression profiles of tRFs – a novel class of small ncRNAs derived from active cleavage of tRNAs – in mouse spermatozoa [44]. tRFs are mainly involved in paternal inheritance and in the inactivation of retroviral elements of the genome. Interestingly, injection of sperm tRF fractions from HFD males into normal oocytes resulted in offspring with altered expression of genes related to metabolic pathways and disorders [44]. In the same issue of Science journal, Sharma and colleagues evaluated how a diet with restricted protein intake can interfere with the expression profile of genes involved in metabolism, as well as small ncRNA biogenesis. Using assisted reproductive techniques (ART), they found that the offspring of fathers with low-protein diet had significant upregulation of genes involved in cholesterol production in the liver. In addition, RNAs from epididymal cauda spermatozoa revealed the presence of an important fraction corresponding to tRFs (28–34 nt), mapping to the 5’end of tRNAs. Notably, the levels of tRNA-Gly-CCC, -TCC, and -GCC were higher in low-protein dietary mice than in controls. In contrast, the Let-7 family of miRNAs was downregulated in low-protein spermatozoa. Also, the analysis of RNA content in different tissues revealed the existence of intense tRNA cleavage in the epididymis but not in the testis, suggesting that tRFs can be released from the epididymis to spermatozoa via the fusion of these cells with small extracellular vesicles (EVs), called epididymosomes [25]. EVs are membranous nanoparticles naturally produced by cells that play an important role in cell-to-cell communication [91, 92]. Indeed, EVs contain nucleic acids (both DNA and RNAs), proteins, lipids, metabolites, etc., that they deliver to target cells [93, 94]. Notably, the epididymosome RNAome has been shown to consist of ⁓87% of tRFs [25]. In particular, tRF-Gly-GCC – upregulated in low-protein dietary spermatozoa – is able to inhibit the expression of genes associated with the retroelements MERVL, with potential effects on preimplantation. Overall, these data demonstrated that paternal diet could influence embryo development via sperm RNAs [25].

One year later, in 2017, Guo and coworkers demonstrated that treatment of mature mouse spermatozoa with lysolecithin, pronase and RNases efficiently removed (⁓90%) sperm-carried RNAs. When the authors used these spermatozoa for ART (i.e., injecting them into normal oocytes by intracytoplasmic sperm injection, ICSI), they found a decrease both in the rate of blastocyst formation and in the live birth rate [36]. Furthermore, even if the offspring born from RNA-depleted spermatozoa developed a normal reproductive capacity, their body weight was lower than the control group, confirming the importance of sperm-carried RNAs for embryo development [36]. In 2018, Zhang and colleagues showed that deletion of *Dnmt2* reduced the levels of m5C and m2G modifications in 30–40 nt ncRNAs in mouse spermatozoa [72]. These modifications were elevated in the sperm RNAs of male HFD mice, thus demonstrating that the deletion of *Dnmt2* prevented the transmission of HFD-induced metabolic disorders to the offspring. Importantly, the deletion of *Dnmt2* was also responsible for altering the expression profile and the secondary structure of small ncRNAs (e.g., tRFs and rRNA-derived small RNAs), supporting the importance of RNA modifications for the preservation of paternal inheritance information [72]. The same year, Conine et al. demonstrated the importance of small ncRNAs acquired by spermatozoa during epididymal transit, for embryo development in mice [38]. The authors generated two different types of zygotes (by ICSI), using spermatozoa from the proximal region of the epididymis (caput) or its distal portion (cauda), and then analyzed the embryo development. Caput spermatozoa generated embryos overexpressing the regulatory factors required for preimplantation development, and did not implant. However, the injection of caudal small ncRNAs into caput-derived embryos completely rescued the preimplantation molecular defects, and resolved the implantation problems [38].

More recently, in 2020, Tyebji and colleagues demonstrated that paternal infections could also alter sperm small ncRNA profiles and consequently offspring behavior [39]. Toxoplasma-infected male mice showed decreased total sperm count and increased sperm morphological abnormalities, which resulted in behavioral changes of F1-F2 offspring, in a sex-dependent manner. Furthermore, toxoplasma infection-induced large differences in the small ncRNA load carried by spermatozoa, with possible implications for the offspring. Of note, zygotic microinjection of small ncRNAs from infected spermatozoa was able to partially recapitulate the behavioral changes observed in the naturally born offspring of Toxoplasma-infected mice [39] (Table 2).

In conclusion, data from animal models support the role of sperm RNAs in early embryo development. These findings require thorough validation in humans.

## Human evidence: impact of sperm epigenetic mechanisms 

In 2004, Marques and colleagues first described the association between oligozoospermia and loss of DNA methylation in humans [57]. The authors investigated in 27 normozoospermic men vs. 96 oligozoospermic patients whether imprinting defects were associated with impairments in spermatogenesis. They extracted the sperm DNA and studied the methylation profiles of two imprinted genes, the *mesodermal specific transcript* (*MEST*), which is maternally imprinted (methylated, repressed) [95], and *H19*, which is instead paternally de novo methylated during the premeiotic phase of spermatogenesis (unmethylated, therefore expressed, in the maternal allele) [96]. They found that the maternal imprint of the *MEST* gene was correctly erased in all samples, while some of the oligozoospermic samples, with reduced sperm motility, showed differential *H19* methylation profiles. Some patients had incomplete methylation and others had a heterogeneous sperm population, half with a hypomethylated allele. In particular, they found hypomethylation at the CTCF-binding site, responsible for the repression of *IGF2* in the maternal allele [57]. Importantly, when they analyzed the methylation profile of the *LINE1* transposon in *H19* hypomethylated patients, they found that the methylation levels were high, confirming that these defects were specific to the imprinted genes [97].

In 2010, Poplinski et al. analyzed the differentially methylated regions (DMRs) associated with *IGF2*/*H19* imprinting control region 1 (ICR1), and with *MEST*, in spermatozoa from 148 idiopathic infertile patients and 33 age-matched normozoospermic controls [78]. *IGF2*/*H19* ICR1 methylation was significantly reduced in oligozoospermic patients (total sperm count < 40 million spermatozoa/mL) vs. controls (89.6% vs. 95.9%, respectively). In contrast, *MEST* was hypermethylated in patients (9.6% vs. 4.3%). In particular, they found that spermatozoa with low-motility had hypomethylation of *IGF2*/*H19* ICR1 and hypermethylation of *MEST*. Also, *MEST* hypermethylation was associated with poor sperm morphology [78].

The same year, Wu and collaborators investigated the association between idiopathic male infertility and the methylation status of the *methylenetetrahydrofolate reductase* (*MTHFR*) gene [46]. *MTHFR* encodes an important enzyme involved in folate metabolism, DNA synthesis and remethylation reactions, with a key role in regulating the balance between DNA synthesis and DNA methylation. Furthermore, this gene is involved in spermatogenesis, as it is highly expressed in mouse testis, and hypermethylation of its promoter is associated with azoospermia [46]. The authors found that 45% of idiopathic patients had *MTHFR* hypermethylation compared with 15% of fertile controls. Interestingly, when they divided idiopathic infertile patients by sperm count, they found that oligozoospermic patients (< 20 million/mL) exhibited higher methylation patterns than normozoospermic men (≥ 20 million/mL). These results confirm that *MTHFR* hypermethylation is associated with idiopathic male infertility and the analysis of its methylation status can be considered a biomarker useful to identify men with a higher risk of infertility [46, 98].

The association between aberrant sperm DNA methylation and low sperm motility was investigated in 2011 by Pacheco et al., who also included the analysis of the sperm RNAs [48]. Their integrated analysis revealed that low-motile spermatozoa exhibited genome-wide DNA hypomethylation, likely due to the failure of chromatin compaction, as revealed also by the high levels of the histone deacetylase HDAC1, which interfere with the histone-to-protamine transition during the spermatogenesis. Also, they speculated that the high production of radical oxygen species (ROS) in low-motile spermatozoa might be due to the decrease of sirtuin 3 (SIRT3) mRNA, with a consequent reduction of the expression of the *antioxidant manganese superoxide dismutase* (*MnSOD*). They suggested that increased ROS production might interfere with the capacity of DNMT3A to identify and set its marks, thus contributing to the hypomethylated phenotype [48].

The same year, Marques and colleagues carried out the analysis of DNMTs – at mRNA and protein levels – in human adult dividing mitotic (spermatogonia A), pre-meiotic (primary spermatocytes), post-meiotic (secondary spermatocytes and round spermatids) and differentiating cells (elongated spermatids/spermatozoa) [58]. The *H19* and *MEST* genes were found to be methylated and demethylated, respectively, in all the stages analyzed. Also, they found that at all the stages DNMT1 expression was higher than DNMT3A/3B, while they did not detect any DNMTs in elongated spermatids. Conversely, at the protein level, DNMT enzymes were present at all stages of spermatogenesis. In particular, the enzymes co-localized: i) in the nucleus of pachytene and secondary spermatocytes, suggesting re-methylation events during meiotic recombination and before the second meiotic division; ii) in the nucleus of elongated spermatids associated with the histone-to-protamine transition to prevent imprinting errors transmittable by the male gamete [58].

Another aspect concerns the association between global methylation and the tendency of human spermatozoa to undergo spontaneous apoptosis. In 2013, Barzideh et al. used HPLC, FC and ICC to detect DNA methylation levels in human spermatozoa from unselected normozoospermic volunteers. They found that low-quality spermatozoa retrieved from the low-density region of the Percoll gradient showed higher levels of 5mC, associated with high levels of annexin V (a marker of early apoptosis). The authors, therefore, suggested that the defective and apoptotic spermatozoa were the result of spermatogenesis disorders leading to hypermethylation of sperm DNA [49].

The process leading to spermatogenesis involves several phases, including epigenetic modifications and the gradual elimination of mitochondrial DNA (mtDNA). Considering that patients with abnormal sperm parameters have higher mtDNA copies than fertile men, Tian and colleagues evaluated the relationship between DNA methylation and mtDNA copy number with human semen quality [99]. In fertile men, sperm DNA was found fivefold more methylated than in somatic cells from blood. In idiopathic infertile patients, *LINE-1* and the maternally imprinted *LIT1* gene were hypomethylated compared to somatic cells, while *H19* was hypermethylated. Also, *LIT1* and *LINE-1* methylation levels were positively associated with those of the testis-specific *BRDT* and the *MTHFR* genes. Computer-assisted parameters of sperm motility were significantly correlated with sperm concentration and morphology, thereby confirming that sperm motility is an indicator of sperm quality. Overall, considering that mtDNA is negatively correlated with these parameters, a low mtDNA copy number can be considered an indicator of sperm quality [99].

In 2017, Laqqan and colleagues deepened the study of different patterns of DNA methylation in 15 infertile patients vs. 15 proven fertile men (with at least two children) [50]. They selected 4 CpG sites (within the genes *PRICKLE2*, *ALS2CR12*, *ALDH3B2* and *PTGIR*) differentially methylated between patients and controls for further validation in 111 samples (55 infertile patients/with abnormal sperm parameters and 56 fertile controls). The data showed a significant difference in the mean methylation levels across all *PRICKLE2* CpGs (lower in patients) and *ALS2CR12* (higher in patients). For *ALDH3B2*, 7 out of 13 CpGs were less methylated in patients, and the same for 9 out of 26 CpGs of *PITGIR*. These variations correlated with the differences in sperm quality between fertile men and infertile patients. Indeed, the latter showed lower values of semen volume, sperm count, motility, vitality and normal morphology [50].

In 2020, Santana et al. analyzed the global DNA methylation pattern in spermatozoa of patients with varicocele, the most common risk factor for male infertility [69]. As expected, sperm concentration, viability and normal morphology were lower in patients than in controls. A genome-wide analysis showed that 54 CpG sites were hypomethylated in patients with varicocele, while 5 were hypermethylated. Also, the authors observed the presence of 1,695 DMRs in genes involved in DNA methylation, gamete generation, piRNA-related processes and meiosis. Interestingly, the regions with increased DNA methylation were associated with H3K27 methylation in the varicocele group, further supporting the potential crosstalk between epigenetic marks. A more accurate analysis pointed out only 24 DMRs with a strong association with genes involved in genetic imprinting and gene expression regulation. Interestingly, these regions were hypermethylated in varicocele patients, without differences between varicocele degrees [69].

As said above, environmental conditions and lifestyle habits may affect male fertility and embryo development. For example, cannabis use decreases sperm concentration and alters DNA methylome, but it is not known yet whether these modifications may be passed to the next generation [32]. As in rodents, significant correlations between aberrant DNA methylation patterns and tobacco smoke were found in humans, with a negative impact on sperm parameters [68]. Dong and colleagues investigated the association between methylation of *H19* and *SNRPN* ICRs with male infertility in cigarette smokers [51]. Interestingly, they found that *H19* hypomethylation and *SNRPN* hypermethylation were strongly correlated with a high risk of infertility, and this risk was potentiated in cigarette smokers [51].

The effects of long exposure to air pollution were also investigated in a recent study by Cheng et al. [52]. DNA methylation and 14 semen parameters were evaluated in 1,554 fertile men, finally classified as normal (⁓62%) and abnormal (⁓38%). The results showed that long-term exposure to single air pollutants (i.e., SO_2_, NO_2_, PM_10_ and PM_2.5_), or the co-exposure to several pollutants, was correlated to reduced total sperm motility, with consequences on time to pregnancy. Interestingly, a sensitivity analysis showed that the association between air pollutants and sperm motility was significant also for non-smokers and non-drinkers [52]. Moreover, analysis of DNA methylation following PM_10_ exposure revealed a positive association with the levels of 5hmC – another type of epigenetic mark recently found in human brain and embryo stem cells, whose precise function is still not fully elucidated – but not with 5mC levels [52].

From the early 2000s, several studies revealed that children conceived with ART have an increased prevalence of imprinting disorders, such as Beckwith-Wiedemann’s (11p15.5), Prader–Willi and Angelman (15q11-q13) syndromes, compared to children conceived naturally [100]. Therefore, the treatment of male infertility may be responsible for the transmission of paternal imprinting errors. Indeed, *H19* hypomethylation leads to the presence of 2 inactive *IGF2* genes, with detrimental consequences for embryo development [57]. The analysis of the global methylation level (GML) of sperm DNA carried out during ART cycles revealed that, differently from the fertilization rate, embryo development may be impaired if GML is below a certain threshold value [101]. Epigenetic alterations associated with ART may depend on the introduction of aberrantly methylated DNA into the zygote by the spermatozoa. In particular, male infertility is significantly associated with defects in the DNA methylation pattern of human spermatozoa. However, it is not clear whether these defects may be ascribed either to hypomethylation, hypermethylation, or both [49].

Finally, a recent systematic review analyzed the association between the methylation of specific genes, sperm DNA fragmentation, and the outcome of pregnancy. The authors reported aberrant methylation of *H19* and *KCNQ1* genes in patients with high sperm DNA fragmentation. Also, a significantly lower H19 methylation rate was found in patients with idiopathic recurrent pregnancy loss and infertile patients, compared to fertile men. Lastly, aberrant *GLT2* methylation was found in patients with poor ART outcomes [102] (Table 3).

In conclusion, several pieces of evidence strongly suggest the presence of aberrant gene methylation in infertile patients. In particular, *H19* hypomethylation appears to increase the risk of pregnancy loss, as well as to affect embryo growth. However, in general, the relationship between the methylation of other genes and ART outcomes, as well as the morphokinetic parameters of this technique, needs to be further investigated.

## Human evidence: impact of sperm RNAs 

Human sperm RNAs were identified starting from 1999, by cDNA cloning and sequencing [103]. Since then, and thanks to the new technologies available for the study of RNAs, such as RNA-seq, the RNAome of male gametes was further characterized [23–25]. However, several RNA populations ascribed to spermatozoa are possibly due to contamination from somatic cells, thus leading to misinterpretation of sperm RNA analyses. Indeed, a single human spermatozoon contains ⁓50 fg of RNA and ⁓0.3 fg of small ncRNAs, which is 200 times less than other cell types [104]. For this reason, the development of new protocols for sperm isolation is crucial for implementing the available information on sperm RNAs.

Several mRNAs encoding for transcription factors, protein kinase, growth factors, etc. have been identified, and some of them were found differentially expressed in infertile patients [104]. Furthermore, the lack of certain sperm mRNAs and/or their specific mutations have been proposed as markers and effectors of male infertility, with a possible function related to the delivery to oocytes [105–107]. These transcripts encode proteins mainly involved in fertilization and embryo development, such as clusterin (CLU) and calmegin (CLGN); or the integrator complex subunit I (INTSI), involved in the early stages of embryogenesis [108]. Sperm transcripts involved in fertilization and post-fertilization have been described as mainly located in genomic regions enriched for H3K4me3, associated with transcriptionally active/poised chromatin. Therefore, these results suggested that sperm chromatin might be able to undergo de novo transcription in mature spermatozoa [24]. These findings highlighted a new key role for spermatozoa, not simply genome carriers, but possibly transcriptionally active cells crucial for embryo development and offspring health [26].

Data from mouse models have shown that the success rate of ICSI is related to the origin of spermatozoa. Spermatozoa obtained from the cauda of the epididymis generate embryos with various genetic regulation problems, while the transit through the epididymis is essential for acquiring small ncRNAs key for fertilization and embryo development [38]. For this reason, the evaluation of some parameters can be useful for assessing ART outcomes [38]. However, experimental evidences in humans do not support data from mouse models. No differences in fertilization and embryo development have been observed with either testicular or caput epididymal spermatozoa in ART cycles, as demonstrated for patients with azoospermia factor c (AZFc), microdeletions and obstructive azoospermia [109, 110]. Interestingly, the most abundant miRNA found in human spermatozoa, miR-34c, was positively correlated with the ICSI success rate by Cuiand co-workers in 2015 [53]. Nevertheless, several children conceived by ICSI had congenital malformations and poor-quality sperm, thus supporting that RNAs acquired during the epididymal transit are important for the intergenerational/transgenerational subfertility passage [111–114]. Indeed, paternally acquired phenotypes (e.g., mental stress and metabolic disorders) can be transmitted to offspring via alterations of sperm small ncRNA levels [72].

Other small ncRNAs found within spermatozoa are piRNAs, involved in the silencing of transposable elements in germline cells, thus protecting the integrity of the genome and playing an important role in spermatogenesis [115]. In obese men, several sperm piRNAs were found altered, whose predicted targets are genes involved in food intake regulation, supporting the role of piRNAs in the inheritance of metabolic disorders [47].

Additional transcripts involved in sperm maturation and fertilization are protamine (*PRM1* and *PRM2*), and transition protein (*TNP2*) [116–118]. Protamines and transition proteins enable proper packing of chromatin during sperm maturation, thereby influencing sperm count, morphology and, of course, sperm functions. For this reason, Savadi-Shiraz and colleagues in 2015 analyzed the levels of PRM1, PRM2 and TNP2 mRNAs in spermatozoa of both normozoospermic men and teratozoospermic patients, based on sperm morphology and ART outcomes [119]. The results showed that PRM1 and PRM2 transcript levels were lower in patients. Furthermore, while the ratio of PRM1 and PRM2 was ⁓1 in controls, it was ⁓10 in teratozoospermic patients [119]. Interestingly, ⁓42% of the latter (30 out of 72) underwent at least one ICSI cycle, and pregnancy (with a live baby) was achieved in 36.6% of couples (11/30). Of these, approximately 70% showed a 1:1 ratio of PRM1 and PRM2, thus supporting the notion that a normal protamine mRNAs ratio is associated with a higher fertilization rate [120]. In contrast, TNP2 levels were higher in the teratozoospermic group, correlated with abnormal sperm head morphology and, in particular, with an increased percentage of round head defect in semen [119]. Also, low protamination was related to sperm tail defects (short tails), and multiple chromatin breaks were found in teratozoospermic patients. These results suggested that PRM1, PRM2 and TNP2 transcripts can be used as biomarkers of fertility and as an index of sperm morphology and fertility [119].

As expected, both environmental toxins and lifestyle cues, such as diet and mental stresses, negatively affect sperm parameters. Tobacco-smoking male partners of couples undergoing ICSI showed higher protamine deficiency and sperm DNA fragmentation, in association with lower expression of *H2BFWT*, *PRM1*, *PRM2*, *TNP1* and *TNP2*. Also, the ratio of protamine mRNAs was higher compared to non-smoking men [55].

In 2020, another pilot study evaluated whether levels of sperm mRNAs encoding proteins with an active role in fertilization, oocyte activation, chromatin remodeling and DNA repair, might differ between oligozoospermic patients undergoing ICSI and controls. A significantly lower level of 21 mRNAs (e.g., AKAP4, PTK7, PLCζ and POU5F1) was reported in patients, whose 14% of oocytes were not fertilized and 90% of the embryos did not reach the morula stage [56]. More recently, a study conducted in 54 normozoospermic men undergoing ICSI for unexplained infertility demonstrated that 324 small ncRNAs (e.g., miR-4755-3p, miR-92a-3p, 5’-tRF-Asp-GTC; 5’-tRF-Phe-GAA, let-7f-2-5p, etc.) were differentially expressed in the samples leading to high vs. low blastocyst formation rate [121] (Table 3).

In conclusion, emerging animal evidence suggests a role for sperm RNAs on sperm parameters, sperm DNA fragmentation, natural conception, pregnancy rate, miscarriage rate and live birth rate in ART programs. This appears to be confirmed by available human studies. However, human data are often scarce and mostly based on a very low number of trials. Therefore, no firm conclusions can be drawn on the mechanistic relationship between sperm RNA levels and embryo kinetics. Importantly, more robust protocols for the isolation of specific germ cell-derived RNAs need to be further developed.

## Conclusions

According to the WHO, the diagnosis of infertility, particularly *sine causa*, in couples attempting to conceive, represents a growing global burden. The prevalence of the male factor is constantly increasing, being associated with numerous sperm abnormalities, as well as problems of embryo development. Nowadays, the decrease in fertility is one of the main factors contributing to a progressive aging of the global population [122, 123]. Therefore, understanding the molecular mechanisms underneath male infertility would help to counteract the aging of the global population and to identify new indicators for effective diagnosis and management of infertility. In this context, how relevant is the role of epigenetics and RNAs in sperm health and embryo viability? Is it a truth or a myth? Research is currently uncovering the multiplicity of functions played by sperm epigenetics and sperm RNAs. Both can be regarded as potential molecular drivers for proper sperm development, as well as for positive fertilization outcomes and viable embryo development.

Overall, the findings described in this comprehensive literature review support the notion that sperm epigenetics, especially sperm DNA methylation patterns, are closely linked to male fertility. Looking at the evidence from animal studies, the exposure to environmental and lifestyle factors, which are often a cause of infertility (cigarette smoke, environmental pollutants, or diet-related diseases), can influence the epigenetic profile of spermatozoa. Furthermore, available animal data suggest the role played by sperm RNAs (coding and non-coding) in embryo development. Specifically, the passage through the epididymis seems crucial to acquire an RNA profile that ensures sperm competence, thus avoiding an embryo-lethal phenotype.

Evidences from human studies further support the occurrence of epigenetic alterations in spermatozoa from patients with abnormal sperm parameters. Similar to animal studies, cigarette or cannabis smoking and exposure to environmental pollutants negatively affect the epigenetic profile of spermatozoa and the health of the offspring. For instance, the hypomethylation of the *H19* gene leads to the presence of two inactive *IGF2* alleles, leading to defective embryo development. Again, sperm RNAs appear to play a role in both post-fertilization events and early-stage embryogenesis. RNAs acquired during epididymal transit are key for intergenerational/transgenerational subfertility. In particular, specific small ncRNAs are involved in paternally acquired phenotypes, such as piRNAs, which are crucial in the inheritance of metabolic disorders.

Gaining a deeper understanding of the epigenetic and RNA landscapes is a crucial step in discovering new factors that contribute to male infertility. The key to obtaining reliable results is choosing the most appropriate technique for sperm isolation and DNA/RNA analyses. In this review, animal studies mainly focused on spermatozoa as the starting tissue to analyze the epigenetic profile or their RNA content. Most of the included human studies adopted reliable, well-known, and standardized protocols for sperm isolation. This includes swim-up, gradient centrifugation, and SCLB incubation, with the first two used in clinical practice for sperm selection in the ART setting. Furthermore, we compared various techniques used to study the epigenetics of DNA and RNA populations, from single locus to genome-wide and transcriptome-wide assays. The range of omics techniques is constantly evolving, to meet the needs of researchers and clinicians. WGBS is considered the gold standard for studying DNA methylation. However, bisulfite treatment is an aggressive chemical method that, despite uncovering the presence of 5mC in CpG-rich sites, significantly reduces sequence complexity with biased genomic coverage. It also neglects other modifications, such as 5hmC. Emerging techniques, using enzymatic conversion of cytosine or third-generation sequencing, are arising to address these biases. Overall, these approaches aim to broaden research to the entire spectrum of chromatin modifications, and their potential functions.

On the other hand, while gene expression analysis has been largely optimized for long RNA species such as mRNAs, the task still requires careful development for small ncRNAs. This is of particular importance to limit biases in reverse-transcription, ligation, library preparation, and to identify reliable housekeeping genes for normalization of small ncRNAs data. Finally, RNA-seq pipelines need more accurate algorithms to identify putative small ncRNAs (e.g., piRNAs, tRFs), amid the vast complexity of genomic data. Overall, these new results may reveal intriguing perspectives on the complex interplay that regulates the sperm genome, from maturation to the early stages of embryo development. This is crucial for the search for the strongest candidate markers in the context of the sperm gene expression program.

In conclusion, multiple lines of evidence point to sperm epigenetics, and in particular sperm DNA methylation, as a relevant factor in the context of seemingly inexplicable male infertility. Regarding sperm RNAs, due to the very low quantity transported by spermatozoa, current protocols for RNA evaluation need to be further optimized. The identification of new potential targets of male infertility and predictors of poor ART outcomes can be used in diagnostic flowcharts of infertile male patients. Importantly, data showing the relationship between sperm epigenetics/RNAs and offspring health can introduce exciting new insights into the counseling of infertile patients. In the near future, the field will need to implement fundamental research with high-quality, well-sized, and adequately controlled studies.

## Data Availability

Not applicable.
